# Gastric Syphilis in a Human Immunodeficiency Virus–Infected Patient

**DOI:** 10.31662/jmaj.2018-0025

**Published:** 2019-02-01

**Authors:** Eisuke Adachi, Tomohiko Koibuchi, Hiroshi Yotsuyanagi

**Affiliations:** 1 Department of Infectious Diseases and Applied Immunology, IMSUT Hospital of The Institute of Medical Science, The University of Tokyo, Tokyo, Japan

**Keywords:** Gastric syphilis, unusual complications of syphilis, Immunostaining, Human immunodeficiency virus

A 35-year-old homosexual man with HIV receiving antiretroviral therapy was admitted to our hospital because of epigastric discomfort that developed three weeks earlier. There were no lesions inside his genital area and no characteristic rash of syphilis. However, gastroduodenoscopy revealed diffuse erosive lesions in the gastric mucosa (Figure 1) and spirochete cells were identified by immunostaining the biopsy specimens (Figure 2). He was diagnosed with syphilis on the basis of the serological test for syphilis. The gastric lesions disappeared after the administration of amoxicillin. We infer that the gastric syphilis was caused by hematogenous dissemination. Some reports have described that intestinal complications are seen in persons who have anal intercourse ^(1)^. Syphilis is a forgotten etiology of active gastritis ^(2)^. Gastric syphilis has become less common, owing to the advances in techniques for the early diagnosis and treatment of syphilis. Immunostaining with anti-*Treponema pallidum* antibodies helps in histologically identifying *Treponema pallidum*.

**Figure 1.**
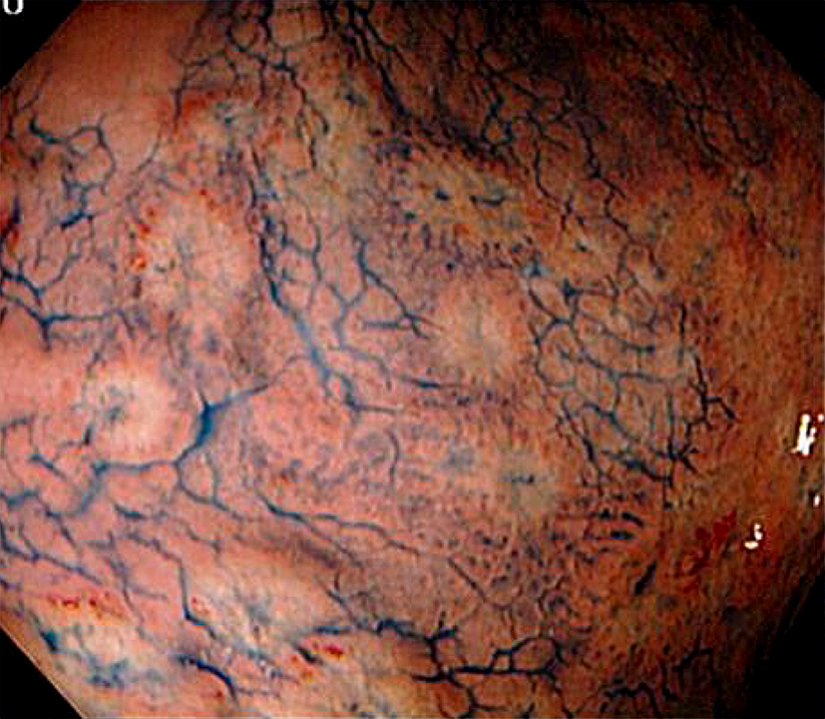
Gastroduodenoscopy showing multiple erosive lesions in the entire gastric mucosa (indigo carmine dye contrast).

**Figure 2.**
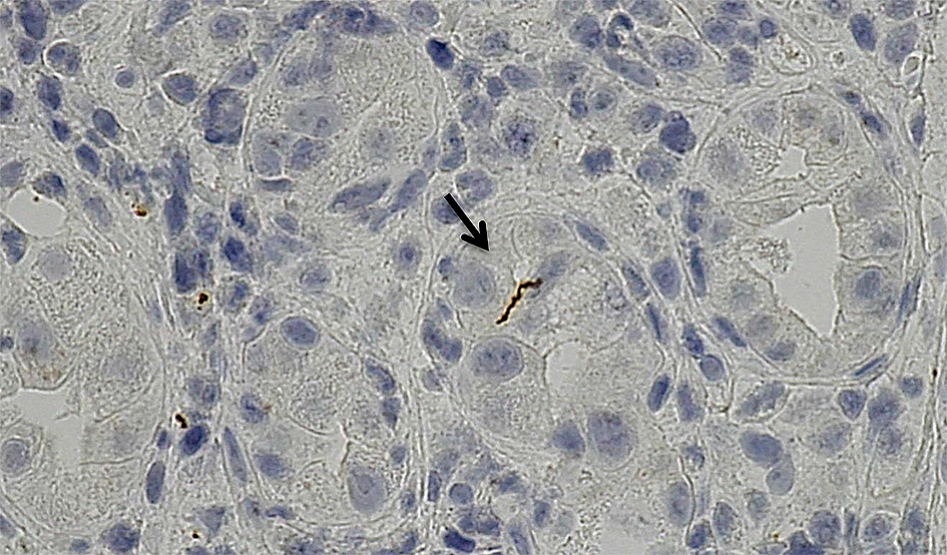
Gastric biopsy specimen immunostained with anti-*Treponema pallidum* polyclonal antibodies showing several spirochete cells stained brown in the interstitium (arrow). Magnification, ×400.

## Article Information

### Conflicts of Interest

None

### Sources of Funding

This work was supported by JSPS KAKENHI grant number 17K16222.

### Author Contributions

EA carried out this work and drafted the manuscript. EA, KT, and HY designed the project and revised the manuscript.

### Informed Consent

A written informed consent was obtained from the patient.

### Approval by Institutional Review Board (IRB)

This work was approved by the Institutional Review Board The Institute of Medical Science, The University of Tokyo (accession number:18-11-1003)
